# Analysis of the Ischemia-Modified Albumin as a Potential Biomarker for Cardiovascular Damage in Obstructive Sleep Apnea Patients with Acute Coronary Syndrome

**DOI:** 10.3390/ijms24109019

**Published:** 2023-05-19

**Authors:** Pilar Resano-Barrio, Enrique Alfaro, Esther Solano-Pérez, Carlota Coso, Carolina Cubillos-Zapata, Elena Díaz-García, Sofía Romero-Peralta, Jose Luis Izquierdo-Alonso, Ferran Barbé, Francisco García-Rio, Manuel Sánchez-de-la-Torre, Olga Mediano, on behalf of the Spanish Sleep Network

**Affiliations:** 1 Sleep Unit, Pneumology Department, Hospital Universitario de Guadalajara, 19002 Guadalajara, Spain; 2 Medicine Department, Universidad de Alcalá, 28805 Madrid, Spain; 3 Centro de Investigación Biomédica en Red de Enfermedades Respiratorias (CIBERES), 28029 Madrid, Spain; 4 Respiratory Diseases Group, Respiratory Service, Hospital Universitario La Paz, IdiPAZ, 28046 Madrid, Spain; 5 Translation Research in Respiratory Medicine, Hospital Universitari Arnau de Vilanova-Santa Maria, IRBLleida, 25198 Lleida, Spain; 6 Pneumology Department, Hospital Universitario La Paz, IdiPAZ, 28046 Madrid, Spain; 7 Faculty of Medicine, Universidad Autónoma de Madrid, 28049 Madrid, Spain; 8 Precision Medicine Group in Chronic Diseases, Respiratory Department, Hospital Universitari Arnau de Vilanova-Santa María, IRBLleida, 25198 Lleida, Spain; 9 Department of Nursing and Physiotherapy, Faculty of Nursing and Physiotherapy, Universidad de Lleida, IRBLleida, 25002 Lleida, Spain

**Keywords:** obstructive sleep apnea, cardiovascular risk, acute coronary syndrome, biomarkers, ischemia-modified albumin

## Abstract

Obstructive sleep apnea (OSA) has been identified as a cardiovascular (CV) risk factor. The potential of OSA promoting the synthesis of CV biomarkers in acute coronary syndrome (ACS) is unknown. Ischemia-modified albumin (IMA) has been identified as a specific CV biomarker. The aim of this study was to evaluate the role of IMA as a potential biomarker for determining the impact of OSA in ACS patients. A total of 925 patients (15.5% women, age: 59 years, body mass index: 28.8 kg/m^2^) from the ISAACC study (NCT01335087) were included. During hospitalization for ACS, a sleep study for OSA diagnosis was performed and blood samples extraction for IMA determination were obtained. IMA values were significantly higher in severe OSA (median (IQR), 33.7 (17.2–60.3) U/L) and moderate (32.8 (16.9–58.8) U/L) than in mild/no OSA (27.7 (11.8–48.6) U/L) (*p* = 0.002). IMA levels were very weakly related to apnea–hypopnea index (AHI) as well as hospital and intensive care unit stay, although they only maintained a significant relationship with days of hospital stay after adjusting for sex, age and BMI (ß = 0.410, *p* = 0.013). The results of the present study would suggest a potentially weaker role of OSA in the synthesis of the CV risk biomarker IMA in patients with ACS than in primary prevention.

## 1. Introduction

### 1.1. Role of Obstructive Sleep Apnea in the Development of Cardiovascular Disease

Obstructive sleep apnea (OSA) is a sleep-related breathing disorder defined as episodes of recurrent upper-airway obstructions during sleep that causes intermittent hypoxia, changes in intrathoracic pressure and sleep fragmentation. The prevalence of OSA has been increasing over time and has been estimated that it might affect 1 billion people worldwide [1]. OSA severity is measured by the number of respiratory events per hour (apnea–hypopnea index (AHI)) considering moderate–severe OSA when the AHI is ≥15 events/h. The most effective treatment for OSA is the continuous positive airway pressure (CPAP) that restores the AHI, improves symptoms and the quality of life of OSA patients [2].

**Figure 1:**
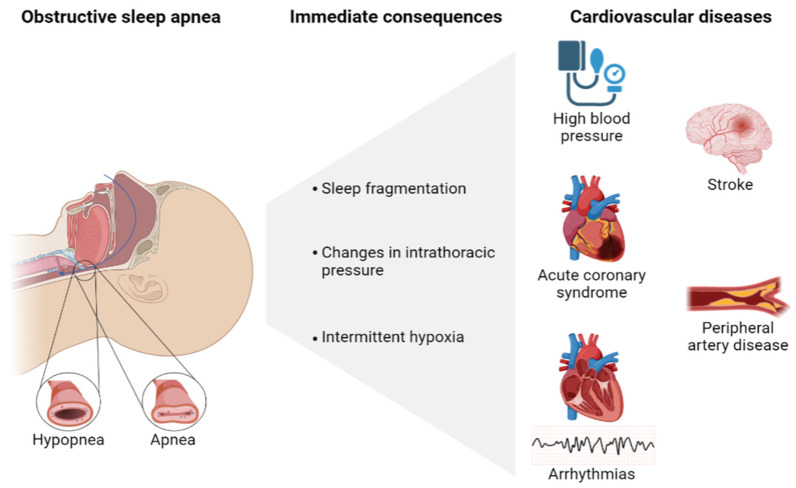
Consequences and cardiovascular diseases implicated in obstructive sleep apnea (OSA). Created with BioRender.

Based on the evidence, OSA can be considered a condition that increases cardiovascular (CV) risk and mortality, including: high blood pressure (HBP), acute coronary syndrome (ACS), arrhythmias, heart failure, stroke and peripheral artery disease (Figure 1). In fact, the prevalence of OSA is higher in patients with CV disease (up to 49%) [3]. The strongest evidence of this relationship is in HBP [4,5] and has been established to have a higher prevalence of OSA (up to 85%) in patients with resistant HBP, with OSA being the most frequent cause of secondary HBP [6]. Additionally, OSA independently increases the risk of coronary artery disease, coronary events [7,8] and has been shown as a possible risk factor for suffering a nocturnal ischemic episode [9]. OSA has also been implicated in an increased risk of CV events after coronary revascularization [10] and with a significant increase in ACS recurrence compared to mild or no OSA [11,12,13].

Whether CPAP therapy prevents major CV events is uncertain. Well-designed randomized controlled studies analyzing the effect of CPAP treatment on CV consequences have not been able to demonstrate the effect of CPAP in CV events prevention [14,15,16,17]. Nevertheless, different studies suggested a potential heterogeneous deleterious role of OSA that would be dependent of some characteristics such as good compliance treatment [14,15], the patient endotype (patients without a previous CV event) [14,18] or the type of the CV event (specifically cerebrovascular) [16] were associated with a better response to treatment. This special response in a certain patient profile suggests that these individuals could be more susceptible to the negative effects of OSA, increasing the risk of developing a new CV event and/or improving the treatment response.

The mechanisms by which some groups of OSA patients would have an increased risk of CV events recurrence and the mechanism implicated are still unknown. It is mandatory to search for specific CV biomarkers for a better understanding of the disease and move toward a personalized medicine in OSA.

### 1.2. Obstructive Sleep Apnea Biomarkers

The measurements obtained in the sleep study establish the severity through the AHI, but this index does not always correlate with the patient’s symptoms and is not predictive of the morbidities associated with OSA. From a pathophysiological point of view, episodes of upper airway obstruction involve a decrease in oxygen saturation (SaO_2_), with intermittent hypoxia followed by reoxygenation that causes sympathetic activation, endothelial dysfunction, hypercoagulability, oxidative stress, inflammation, and metabolic dysregulation [19,20]. Chronic inflammation, as well as oxidative stress, measured through blood biomarkers, play a very important role in diseases associated with OSA, such as CV diseases. In patients with OSA, an increase in blood biomarkers has been described as inflammation biomarkers (C-reactive protein (CRP) and tumor necrosis alpha factor (TNF-alpha)) [21,22], inflammatory cytokines (interleukin 6 (IL-6), interleukin 8 (IL-8)) [21], endothelial dysfunction markers (nitric oxide) [23], hypercoagulability disorders (D-dimer, tissue factor, thrombin and antithrombin levels) [24] and oxidative stress markers (reactive oxygen species (ROS)) [25].

However, none of these biomarkers have been shown to be useful in the identification of patients with OSA, the determination of its severity, or to have the ability to predict the prognosis or response of treatment to date. For this reason, investigations into new biomarkers, with true clinical utility, are required. Recently, a new CV biomarker, ischemia-modified albumin (IMA) has been postulated as a better predictor of disease implications than classical biomarkers [26].

**Figure 2:**
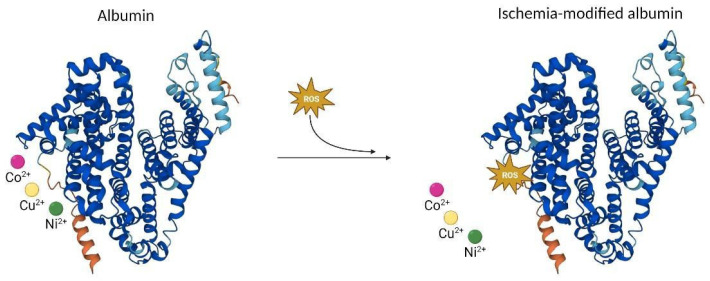
Structure of human ischemia-modified albumin (IMA). Albumin binds transport metal ions such as copper, cobalt and nickel in its N-terminal region. During an ischemic episode, an increase in the oxidative stress species (ROS) occurs, modifying this region and giving rise to the IMA, which reduces the binding capacity of the ions. Co: cobalt; Cu: copper; Ni: nickel; ROS: reactive oxygen species.

Albumin is the protein with the highest plasma concentration, and among its functions is the transport of metal ions, with a high affinity for copper, cobalt and nickel in its amino terminal region (N-terminal). When there is ischemia, a change in the N-terminal occurs that reduces this binding capacity, with ROS probably being the causative agent of this change, giving rise to IMA [27,28] (Figure 2). This change allows us to determine IMA concentration using the albumin cobalt binding test, which identifies colored complexes from unbound cobalt ions [29].

IMA is a new blood biomarker accepted by the Food and Drug Administration (FDA, USA) for the diagnosis of ACS in adults [30,31] as a direct measurement of oxidative stress, an intermediate mechanism involved in the development of arteriosclerosis and CV disease. IMA has been considered a biomarker for the increased risk of CV disease [32,33,34]. Kazanis et al. found that IMA levels were associated with an increased risk of coronary disease (OR = 1.23, 1.16–1.31, *p* < 0.001) and established that the best cut-off point of the IMA for this condition was 101.5 kU/L (sensitivity and specificity of 87.7% and a negative predictive value of 83.3%) [35].

IMA has been postulated to increase the diagnostic sensitivity of conventional cardiac biomarkers. Sinha et al. determined the levels of IMA (in addition to myoglobin, creatine kinase isoform 2 (CK-MB), troponin I (TnI)) in patients with suspected ischemia [36]. The combination of myoglobin, CK-MB and TnI provided a diagnostic sensitivity of 57%, whereas, when they were evaluated in combination with IMA sensitivity, increased to 97% with a negative predictive value of 92%. Gurumurthy et al. detected higher IMA serum concentrations in ACS patients compared to healthy subjects, with a positive predictive value of 96%, a specificity of 80% and a sensitivity of 70%, suggesting a potential role of this protein as a biomarker of ACS [37]. Mehta et al. found higher levels of IMA and cardiac troponin T (hs-cTnT) in patients with ACS compared to controls, indicating that it may be useful for the risk stratification of ACS and a better accuracy in the early diagnosis [38]. Finally, Shin et al. found that patients with unstable angina had greater serum IMA values than patients with non-ischemic chest pain admitted to the emergency department with symptoms suggestive of ACS. The sensitivity of serum IMA was estimated to be 0.74 and the specificity, 0.40 [39].

It could be plausible that the repeated desaturation–resaturation events produced by OSA create a state of ischemia, which influences the albumin N-terminal region. Oxidative stress is one of the demonstrated intermediate mechanisms involved in OSA and is likely responsible for CV damage caused by the illness, but the implicated mechanism has not been completely elucidated to this day [40].

To date, there are only a few studies that correlate OSA and IMA levels (Table 1), having described high levels of it in patients with OSA that could normalize after treatment with CPAP [41,42,43,44,45]. Yang LX et al. [41] were the first researchers investigating the relationship between IMA and OSA. The authors reported that elevated IMA levels significantly associated with OSA and positively correlated with the AHI (r = 0.462, *p* = 0.008). The author determined that the optimal diagnostic cut-off for the estimation of OSA was 54.00 U/L, and the levels had a significant response after CPAP treatment. These results were later confirmed by other authors [42,43,45] who found higher IMA levels in patients with OSA when compared with control, and positively correlated with OSA severity (AHI), but also with body mass index (BMI), being higher in obese patients with OSA relative to non-obese patients. However, Ozben et al. did not find a significant relation between IMA and OSA [40]. In all of these studies, patients with CV risk or disease were excluded. Uygur F et al. [43] also evaluated the impact of CPAP on circulating IMA concentrations in patients with OSA. They found significantly higher serum IMA concentrations in the OSA group than in the control group at baseline, and after three months of CPAP treatment, OSA patients had significantly lower serum IMA concentrations (0.555 ± 0.062 absorbance units (ABSU) to 0.431 ± 0.063 ABSU, *p* < 0.001). This response to CPAP treatment was confirmed by other authors in different studies [44,46].

In the evaluation of IMA levels in OSA patients with CV consequences, carotid intima media thickness has been evaluated, being significantly higher in the OSA group and positively correlated with the AHI and oxygen desaturation index (ODI). These results reflect that IMA, as an oxidative stress biomarker, is related to an increased risk of atherosclerosis production [47].

Taking into account all this evidence, the aim of the present study was to evaluate the role of IMA as a potential biomarker for determining the impact of OSA in ACS patients. We tried to identify other factors associated with ACS severity implicated in this association.

## 2. Results

### 2.1. Patient Characteristics

A total of 925 patients with OSA and ACS, from the ISAACC study (Impact of sleep apnea syndrome in the evolution of acute coronary syndrome. Effect of intervention with CPAP) (NCT01335087) were included. For the whole sample, the mean age of the population was 59 ± 10 years with a mean BMI of 28.8 ± 4.6 kg/m^2^ and 15.5% were female. Excessive daytime sleepiness (EDS) measured by the Epworth sleepiness scale (ESS) was 5 ± 3. According to the sleep study, patients were divided into three groups based on OSA severity (AHI): non-OSA/mild OSA (27.6%), moderate OSA (32.4%) and severe OSA (40.0%). The mean AHI registered was 28.50 ± 21.61 events/h (non-OSA/mild OSA: 6.11 ± 3.97; moderate OSA: 21.38 ± 4.50; severe OSA: 49.70 ± 17.42). Nocturnal SaO_2_ parameters were reduced, including mean SaO_2_ (92.2 ± 7.5%) and lowest SaO_2_ (82.4 ± 10.0%), related to OSA severity (Table 2). Moreover, similar comorbidities were present in the three groups except for HBP, which was more prevalent in severe patients and all groups received similar pharmacological treatment, except for calcium antagonist and beta blockers. The patients’ characteristics, pharmacological treatment and sleep study parameters of the included participants specified by OSA severity are presented in Table 2. Blood sample variables are shown in detail in Table A1.

### 2.2. Cardiovascular Parameters Related to the Severity of Acute Coronary Syndrome

The analysis of the parameters related to the severity of the ACS among groups showed similar results with a mean number of coronary vessels affected as determined by a catheterism procedure of 1.75 ± 0.92 with a mean degree of obstruction of 87.10 ± 20.17%. Related to the treatment procedures needed by the patients in relation to the ACS, a mean of 85.1% of the patients required a stent implantation with a mean number of stents of 1.41 ± 1.14. A mean of 4.7% of the sample required a by-pass surgery procedure as treatment. The mean intensive care unit (ICU) stay was 2.21 ± 1.76 days. However, differences between groups were found in the left ventricular ejection fraction (LVEF), with a mean result of 54.94 ± 10.11% (non-OSA/mild OSA: 57.51 ± 9.36; moderate OSA: 53.21 ± 10.81; severe OSA: 54.59 ± 9.70; *p* < 0.001) and hospital stay (non-OSA/mild OSA: 5.98 ± 2.87; moderate OSA: 7.01 ± 5.17; severe OSA: 6.66 ± 4.29; *p* = 0.017). The parameters related to the severity of ACS of the included participants specified by OSA severity are presented in Table 3.

### 2.3. Serum Levels of Ischemia-Modified Albumin Are Increased in Patients with Moderate–Severe Obstructive Sleep Apnea

**Figure 3:**
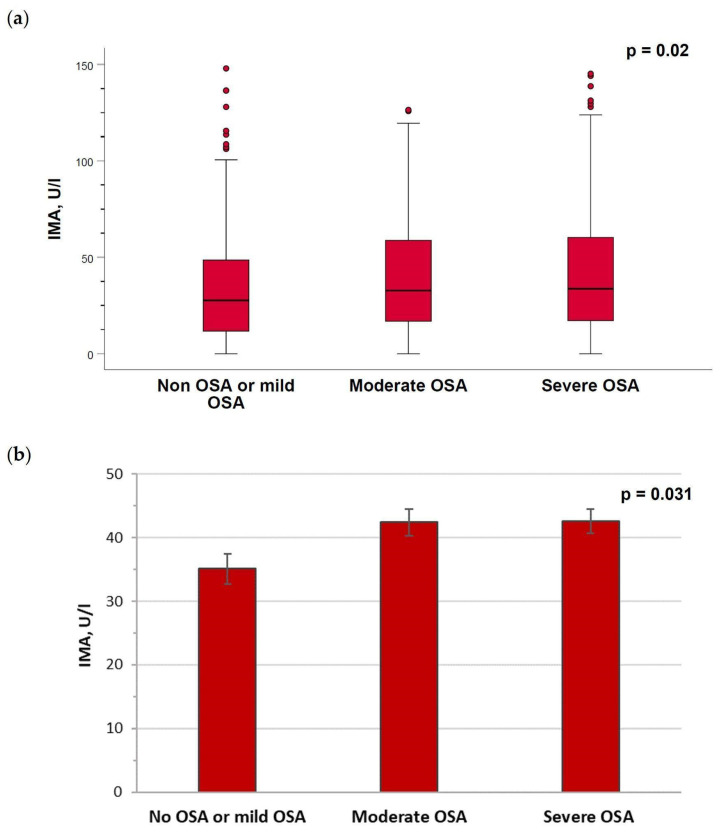
Serum levels of IMA in the study groups. (**a**) Box-and-whisker plots depicting the distribution of serum levels of IMA according to the obstructive sleep apnea (OSA) severity groups corresponding to unadjusted analysis. Data are presented as median (IQR), maximum and minimum values, and overall comparisons were performed using the Kruskal–Wallis test. (**b**) IMA levels when the analysis was adjusted by gender, age, body mass index, neck circumference and waist/hip ratio in the three study groups. Columns correspond to the adjusted mean and the error bars represent the standard error of the mean of between-group comparisons by ANOVA.

IMA levels were higher in patients with severe (median (IQR), 33.7 (17.2–60.3) U/L) or moderate (32.8 (16.9–58.8) U/L) OSA compared with non-OSA/mild OSA (27.7 (11.8–48.6) U/L) (*p* = 0.002) (Figure 3a) in the unadjusted analysis. When the analysis of the IMA values was performed, adjusted by gender, age, BMI, neck circumference and waist/hip ratio, the levels remained higher in severe (adjusted mean ± SEM, 42.6 ± 1.9 U/L) or moderate (42.4 ± 2.1 U/L) OSA patients versus non-OSA/mild OSA patients (35.1 ± 2.4 U/L) (*p* = 0.031) (Figure 3b).

### 2.4. Relation between Ischemia-Modified Albumin Levels and Sleep Parameters

**Figure 4:**
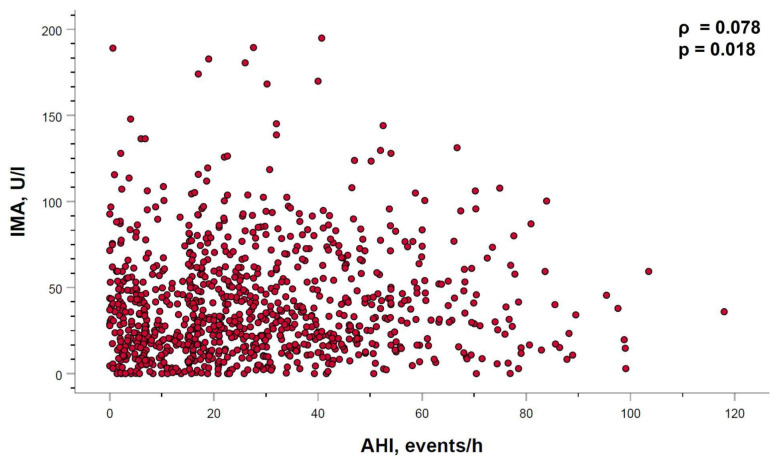
Relationship between serum levels of IMA and apnea–hypopnea index (AHI). Spearman’s correlation coefficient (ρ) and *p*-value sare shown.

In the overall study subjects, serum IMA levels showed a very weak but statistically significant positive correlation with the AHI (ρ = 0.078, *p* < 0.018) (Figure 4). However, when the AHI–IMA relationship was adjusted for age, sex and BMI, statistical significance disappeared (adjusted regression coefficient (ß): 0.066; 95% CI: −0.038 to 0.171; *p* = 0.213). No other relationship between IMA levels and the remaining evaluated sleep parameters were identified.

### 2.5. Ischemia-Modified Albumin Levels Correlation with Cardiovascular Parameters

**Figure A1:**
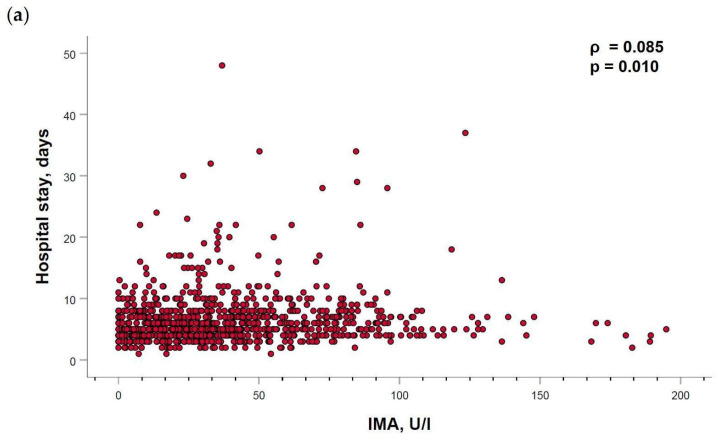

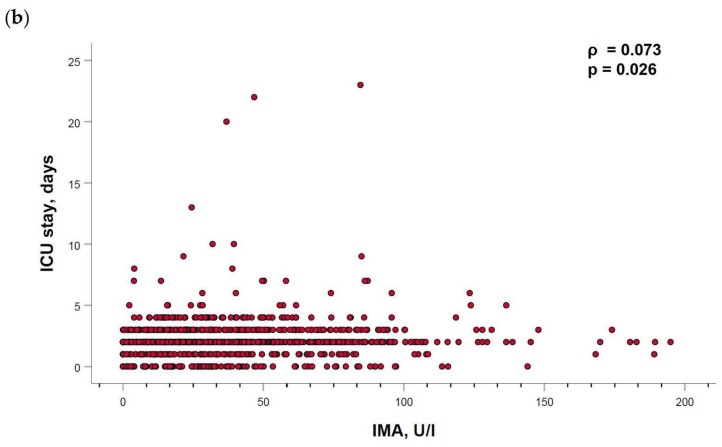
IMA levels correlation with hospitalization. Relationship of serum levels of IMA with (**a**) hospital and (**b**) intensive care unit (ICU) stay in patients with OSA and acute coronary syndrome. Spearman’s correlation coefficients (ρ) and *p*-values are provided.

IMA showed a weak positive significant correlation with the days of hospital stay (ρ = 0.085, *p* = 0.01) and the days needed in the ICU (ρ = 0.073, *p* = 0.026) (Figure A1). Interestingly, the relationship between IMA levels and days of hospital stay was maintained after adjusting for age, sex and BMI (adjusted regression coefficient (ß): 0.410; 95% CI: 0.001 to 0.907; *p* = 0.013). However, IMA values were not correlated with the rest of severity CV parameters such as LVEF, number of coronary vessels affected, degree of coronary obstruction, type of treatment procedures needed (stent implantation or by-pass surgery), history of previous ACS nor the Killip scale.

## 3. Discussion

The results of the present study report, for the first time, the potential role of OSA promoting the synthesis of IMA biomarkers in patients with an ACS. This result indicates that in patients admitted with an ACS, OSA is weakly associated with increased levels of IMA, suggesting a smaller potential implication of OSA as a CV risk factor in this type of patients.

Only a few of the previous studies showed results in OSA patients, with smaller samples, and excluding patients with CV disease. Düger et al. [26], in a retrospective study in 86 OSA patients, showed that serum IMA levels were significantly higher in patients than controls (*p* = 0.008). The level increased significantly with OSA severity (r = 0.50, *p* < 0.001), with results that were consistent with previous studies [41,42,43]. However, some other studies did not find different levels of IMA between OSA patients and controls [40]. These previous existing studies excluded patients with CV risk and/or disease as it is a very frequent condition in OSA patients. To the best of our knowledge, the impact of OSA severity in IMA levels of patients with ACS has not been previously analyzed, and our results establish for the first time this association.

Importantly, the identified association of IMA levels with OSA severity groups in the present study remained significant when adjusted for confounding factors such as age, sex, BMI, neck circumference and waist/hip ratio. These results would indicate the potential relationship between OSA severity and this CV risk biomarker and point to a possible pathophysiological pathway in relation to OSA/CV risk. OSA has been related with an increased CV morbidity and mortality, with oxidative stress as one of the most important mechanisms proposed. The AHI reflects repetitive episodes of hypoxia and reoxygenation experienced during sleep, resulting in the increased production of ROS, responsible for N-terminal albumin modification. These results indicate that IMA may act as a biomarker for oxidative stress in patients with ACS and concomitant OSA.

It is important to highlight that the previous analysis from the ISAACC cohort did not demonstrate a deleterious effect of OSA in CV recurrence. Nevertheless, specific endotypes of patients with OSA and ACS would be vulnerable to OSA increasing the risk of CV recurrence [18]. These results manifest a potential heterogeneously deleterious effect of OSA that would be dependent on the patient’s profile [48]. Moreover, a previous analysis from the ISAACC study evaluated the influence of OSA on the severity of ACS and a short-term prognosis [49], concluding that OSA is associated with an increased ACS severity and length of stay in the ICU. The result of IMA evaluation of the present study would suggest a possible deleterious effect of OSA in the expression of this biomarker of CV disease. Additionally, these results could have an important interpretation in the negative results of RCT, exploring the effect of CPAP in the prevention of CV events or mortality. If OSA has weaker deleterious CV effects in ACS patients, this could explain the negative results. In the ISAACC [17] and SAVE [16] studies, all the participants had previous CV illness. This hypothesis could suggest a possible positive effect of OSA treatment as a primary prevention tool in CV risk prevention.

Another important aspect is the lack of correlation between the IMA and the respiratory sleep parameters. Currently, it has been suggested that the classical parameters that define OSA severity would be insufficient for the identification of associated CV morby-mortality. A new and promising parameter, the hypoxic burden, has recently been described, with a better association to CV risk than AHI in OSA patients [50]. This parameter explores not only the number and frequency of events/desaturation, but also the depth and length of the desaturation. These results would encourage exploring the value of these new variables in the severity and consequences of OSA. Other authors suggest investigating different pathophysiological pathways (dysbiosis, alterations in the autonomic nervous system) responsible for increasing CV risk in these patients [51].

Some study limitations need to be commented on. Firstly, in relation to the timing of the blood sample extraction, it has been suggested by some authors [52] that IMA could have a dynamic ischemic damage in OSA with regard to anoxia produced during the night. This hypothesis has not yet been confirmed, but could be relevant in IMA determination in OSA patients. If there exists a dynamic change in IMA concentrations in OSA patients, this point could help determine the results. However, IMA increases within minutes after the onset of ischemia until 6–12 h [53]. Blood samples in our study were drawn early in the morning, so were properly timed to yield positive results. Secondly, since the results of the present study have been performed in a population of patients with ACS, the present results cannot be generalized to other profiles of patients with OSA. Thirdly, in the ISAACC study, patients with EDS were excluded. Nevertheless, the number of patients excluded for this cause were relatively low and the sample is representative of the phenotype of patients with OSA and ACS. Fourthly, women are underrepresented in this sample, with a predominance of male subjects. Finally, the sleep study for the OSA diagnosis was respiratory polygraphy, which could underestimate OSA severity. However, the effect of OSA in IMA values is most likely determined more by the desaturation than the arousability, and this underestimation is probably slight.

The strengths of the study comprise the large number of samples collected in a well-designed randomized clinical trial in an OSA and ACS multicenter study from a large number of patients. Additionally, all participating centers performed the same blood process methodology and the sleep study was performed with the same model of polygraph. All the variables were collected systematically.

## 4. Materials and Methods

### 4.1. Participants

This is an ancillary blood sample analysis of the ISAACC study: Impact of sleep apnea syndrome in the evolution of acute coronary syndrome. Effect of intervention with CPAP. ISAACC is a multicenter, open-label, parallel, prospective, randomized and controlled trial (registration number: NCT01335087) of OSA with ACS [17]. The 925 patients were hospitalized for ACS at the time of the inclusion of the study in 15 different coronary units or cardiology wards around Spain. They were patients without EDS with an ESS < 10. The inclusion and exclusion criteria and details of the ISAACC study’s method has been previously reported in a previous publication [54].

### 4.2. Procedures

#### 4.2.1. General Procedures

The demographic (age and sex) and anthropometric characteristics (BMI, neck and waist/hip ratio), medical history of chronic disease and usual pharmacological treatment were recorded systematically by a sleep specialist before the sleep study. The degree of EDS was calculated using validated questionnaires to this aim (ESS) [55]. Seated BP was determined from two replicate measurements obtained at least 1 min apart.

#### 4.2.2. Sleep Study Methodology

For OSA diagnosis, all patients underwent a respiratory polygraphy (Embletta, ResMed, Bella Vista, NSW, Australia) in the first 24–72 h after admission, with a register of nocturnal respiratory parameters. Airflow and snoring were measured using a nasal transducer; thoracic and abdominal movements using inductive plethysmography. Blood oxygen saturation and heart rates were measured by digital pulse oximetry and position was recorded by actigraphy. Respiratory events were manually scored, and the criteria for OSA diagnosis and severity were established according to the national guidelines [56]. OSA severity was defined by AHI: mild ≥ 5 events/h and <15/h; moderate ≥ 15 events/h and <30; and severe ≥ 30 events/h.

#### 4.2.3. Blood Sample Determination

For the current post hoc study, to identify the IMA values, a blood sample test was performed the morning after the sleep study. Overnight fasting venous blood samples were obtained from all participants. EDTA (ethylene diamine tetra-acetic acid) anticoagulant tubes were centrifuged (1500× *g* for 10 min at 4 °C) to separate the serum fraction. All specimens were immediately aliquoted, frozen and stored in a dedicated −80 °C freezer until used in the assay. Serum IMA was measured using the human IMA ELISA (enzyme-linked immunosorbent assay) (CSB-E09594h; Cusabio, Houston, TX, USA). Measurements for serum samples were performed in duplicate. The detection limits of the assays were 3.12 IU/mL, while intra-assay and inter-assay variations were below 20%.

All patients underwent fasting blood extraction tubes without anticoagulant, EDTA and citrate. Measures determined were as follows: hemogram, basic biochemical profile (glucose, triglycerides, total cholesterol, LDL cholesterol, HDL cholesterol, uric acid, creatinine, AST, ALT).

#### 4.2.4. Cardiovascular Parameters

Parameters related to ACS severity were reported systematically. Echocardiographic evaluation and Killip classification were routinely performed during patient admission. Other parameters, such as the existence of a previous ACS, parameters derived from the coronarography (number of arteries affected, level of coronary obstruction) and the date related with the procedure needed (stent or surgery and/or number of stents needed) were registered. The hospital stay and the days spent in the intensive care unit were also noted.

### 4.3. Statistical Analysis

Data are expressed as mean ± standard deviation (SD), median (IQR) or absolute numbers and percentages, according to the type and distribution of the variables. Normality was explored using the Shapiro–Wilk and skewness–kurtosis tests. Differences between groups were analyzed using the chi-squared test or Fisher’s exact test (categorical variables: using the second when the expected values in any of the cells of a contingency table were below 5 or below 10 when there was only one degree of freedom), ANOVA (normal variables) and the Kruskal–Wallis test (ordinary or non-normal metric variables). For between-group comparisons, IMA levels were adjusted by gender, age and body mass index using general linear models. The relationship between IMA levels with sleep parameters and clinical or biochemical variables were evaluated using Spearman’s correlation. All tests were two-tailed and a statistical significance level of 0.05 was retained. Analyses were performed using the Statistical Package for the Social Sciences (version 26.0; SPSS, Chicago, IL, USA).

## 5. Conclusions

IMA emerges as a biomarker that would contribute to identify a smaller deleterious CV effect of OSA when ACS is present. This could be related with a possible pathophysiological pathway in relation to oxidative stress produced by OSA, responsible for the albumin modification. In addition, said results could suggest a possible different beneficial effect of OSA treatment between primary and secondary CV risk prevention. New studies should deepen the identification of useful biomarkers for the characterization of CV damage associated with ACS–OSA patients. The identification of new variables related to OSA severity on a better correlation with CV risk could improve the identification of these types of biomarkers.

## Figures and Tables

**Table 1:** Main characteristics and results of studies, including relationship between IMA levels and OSA.

Author (Year)	Number of Participants (OSA/Controls)		Age (Years) and Sex (% Males) (OSA/Controls)	Diagnostic Criteria for OSA Severity	IMA Values (OSA/Controls)	Main Results
Yang LX et al. (2013) [41]	30/32		40/40 69/70%	AHI ≥ 5 events/h	65.10 ± 9.84/ 47.50 ± 9.16 U/L	IMA levels were significantly higher in the OSA group compared with the control group. The CPAP treatment decreased the IMA levels. There was a positive correlation between IMA values and AHI (r = 0.462, *p* = 0.008) at baseline.
Ozben S et al. (2014) [40]	59/25		51/49 56/64%	AHI ≥ 5 events/h	30.30 ± 29.02/ 31.50 ± 48.43	Plasma IMA levels were not statistically different between the OSA patients and controls.
Dogan D et al. (2016) [42]	39/12		41/40 100/100%	AHI ≥ 5 events/h	0.36 ± 0.04/ 0.89 ± 0.14 U/mL	IMA levels were significantly higher in the OSA group compared with the control group (r = 0.600, *p* < 0.05) Mean AHI and IMA values were higher in obese + OSA group.
Uygur F et al. (2016) [43]	97/30		49/52 70/71%	AHI ≥ 5 events/h associated with symptoms or AHI ≥ 15 events/h	0.518 ± 0.09/ 0.415 ± 0.07 ABSU	OSA is associated with elevated concentrations of IMA, which can be reversed by effective CPAP treatment. IMA concentrations correlated significantly with the AHI (r = 0.471, *p* < 0.001).
Sunnetcioglu A et al. (2016) [45]	Mild/moderate OSA Severe OSA No OSA	29 28 22	46/76% 47/75% 36/82%	AHI ≥ 5 < 30/h AHI ≥ 30/h AHI < 5/h	1.98 ± 1.43 U/L 2.28 ± 1.00 U/L 0.84 ± 0.49 U/L	IMA levels were higher in the OSA group compared with controls.
Karamanli H et al. (2016) [47]	61/24		51/49 62/63%		1.23 ± 0.10/1.09 ± 0.16 ABSU	Serum IMA levels were significantly higher in individuals with OSA compared with the control group. IMA levels were positively correlated with the AHI (r = 0.32, *p* = 0.004) at baseline.
Xu et al. (2017) [44]	33/30		52/49 70/70%	AHI ≥ 5 events/h associated with symptoms or AHI ≥ 15 events/h	0.58 ± 0.11/0.43 ± 0.09 ABSU	The levels of IMA were significantly higher in patients with OSA than in the control group. These values can be reversed by CPAP treatment. A significant correlation was noted between IMA and AHI (r = 0.614; *p* < 0.001).
Varikasuvu S.R et al. (2019) [46] Meta-analysis	411/216			AHI ≥ 5 events/h associated with symptoms or AHI ≥ 15 events/h		OSA patients showed a significantly increased IMA level compared with the control group. These IMA values decreased with CPAP treatment Meta-analysis of correlations showed significant associations of IMA with AHI (r = 0.448, *p* < 0.001).
Düger M et al. (2021) [26]	86/83		45/43 63/59%	AHI ≥ 5 events/h	0.46 ± 0.1/0.31 ± 0.09 ABSU	IMA levels were significantly higher in patients with OSA than controls. The serum IMA levels increased significantly as OSA severity increased and was positively correlated with the AHI (r = 0.41, *p* < 0.001).

Abbreviations: OSA: obstructive sleep apnea; IMA: ischemia-modified albumin; AHI: apnea–hypopnea index; CPAP: continuous positive airway pressure. ABSU: absorbance units.

**Table 2:** General characteristics, pharmacological treatment and sleep study parameters of the included participants specified by OSA severity.

Parameters	Non-OSA/Mild OSA (n = 255)	Moderate OSA (n = 300)	Severe OSA (n = 370)	Total (n = 925)	*p*-Value
General characteristics					
Women	38 (14.9%)	56 (18.7%)	49 (13.2%)	143 (15.5%)	0.149
Age, years	58 ± 12	59 ± 10	60 ± 10	59 ± 10	0.199
BMI, kg/m^2^	27.0 ± 3.9	28.4 ± 4.0	30.5 ± 4.8	28.8 ± 4.6	<0.001 *
Neck circumference, cm	40 ± 4	40 ± 4	41 ± 3	41 ± 4	<0.001 *
Waist/hip ratio	0.98 ± 0.07	0.98 ± 0.10	0.99 ± 0.07	0.99 ± 0.08	0.048 *
Sleep parameters					
ESS	5 ± 3	5 ± 2	5 ± 2	5 ± 3	<0.001 *
Study time, min	414.40 ± 58.45	406.78 ± 51.24	408.14 ± 56.42	409.42 ± 55.40	0.232
AHI, events/h	6.11 ± 3.97	21.38 ± 4.50	49.70 ± 17.42	28.50 ± 21.61	<0.001 *
ODI 4%, events/h	9.06 ± 15.34	19.99 ± 14.03	46.76 ± 39.83	27.65 ± 31.96	<0.001 *
Mean SaO_2_, %	92.55 ± 8.42	92.29 ± 7.66	91.92 ± 6.72	92.22 ± 7.52	0.586
Lowest SaO_2_, %	85.05 ± 10.61	82.93 ± 8.55	80.14 ± 9.34	82.40 ± 9.66	<0.001 *
Medical history					
HBP	107 (42.0%)	160 (53.3%)	217 (58.6%)	484 (52.3%)	<0.001 *
Diabetes	55 (21.6%)	73 (24.3%)	91 (24.6%)	219 (23.7%)	0.647
Dyslipidemia	134 (52.5%)	179 (59.7%)	213 (57.6%)	526 (56.9%)	0.226
CV disease	56 (22.0%)	59 (19.7%)	71 (19.2%)	186 (20.1%)	0.679
Cerebrovascular disease	9 (3.5%)	8 (2.7%)	10 (2.7%)	27 (2.9%)	0.793
Respiratory disease	17 (6.7%)	12 (4.0%)	25 (6.8%)	54 (5.8%)	0.255
Neurological disease	18 (7.1%)	14 (4.7%)	13 (3.5%)	45 (4.9%)	0.126
Medication					
ARAII	32 (12.6%)	45 (15.0%)	69 (18.6%)	148 (15.8%)	0.113
ACEI	56 (22.0%)	60 (20.0%)	82 (22.2%)	198 (21.4%)	0.764
Hypolipidemic	96 (37.6%)	117 (39.0%)	140 (37.8%)	353 (38.2%)	0.935
Oral antidiabetic	47 (18.4%)	53 (17.7%)	75 (20.3%)	175 (18.9%)	0.675
Insulin	13 (5.1%)	16 (5.3%)	24 (6.5%)	53 (5.7%)	0.716
Antiplatelet agents	56 (22.0%)	76 (25.3%)	79 (21.4%)	211 (22.8%)	0.441
Anticoagulants	13 (5.1%)	12 (4.0%)	17 (4.6%)	42 (4.5%)	0.824
Beta blockers	59 (23.1%)	69 (23.0%)	58 (15.7%)	186 (20.1%)	0.023 *
Calcium antagonists	19 (7.5%)	39 (13.0%)	60 (16.2%)	118 (12.8%)	0.005 *
Diuretics	36 (14.1%)	49 (16.3%)	76 (20.5%)	161 (17.4%)	0.096
Antacids	65 (25.5%)	74 (24.7%)	96 (25.9%)	235 (25.4%)	0.930

Data are n (%) or mean ± standard deviation. Comparisons between groups by ANOVA or chi-squared tests referring to the global distribution of each variable in the three study groups. * Significant *p*-values (*p* < 0.05). OSA: Obstructive sleep apnea; BMI: body mass index; ESS: Epworth sleepiness scale; AHI: apnea–hypopnea index (number of events/h); ODI: oxygen desaturation index; mean SaO_2_: mean nocturnal oxygen saturation; SaO_2_: oxygen saturation; HBP: high blood pressure; CV: cardiovascular; ARAII: angiotensin II receptor antagonists; ACEI: angiotensin convertase enzyme inhibitors.

**Table 3:** Cardiovascular scales and parameters related to the severity of acute coronary syndrome of the included participants specified by OSA severity.

Parameters	Non-OSA/Mild OSA (n = 255)	Moderate OSA (n = 300)	Severe OSA (n = 370)	Total (n = 925)	*p*-Value
ACS parameters					
LVEF, %	57.51 ± 9.36	53.21 ± 10.81	54.59 ± 9.70	54.94 ± 10.11	<0.001 *
Number of coronary vessels, mean	1.66 ± 0.91	1.77 ± 0.87	1.79 ± 0.95	1.75 ± 0.92	0.201
Vessel obstruction, %	86.78 ± 20.90	86.48 ± 18.41	87.77 ± 20.80	87.10 ± 20.17	0.753
Stent, % of patients	215 (84.3%)	261 (87.0%)	311 (84.1%)	787 (85.1%)	0.523
Number of stents, mean	1.35 ± 1.12	1.40 ± 1.08	1.45 ± 1.21	1.41 ± 1.14	0.563
Surgery, % of patients	6 (2.4%)	16 (5.4%)	21 (5.8%)	43 (4.7%)	0.111
Hospital stay, days	5.98 ± 2.87	7.01 ± 5.17	6.66 ± 4.29	6.59 ± 4.29	0.017 *
ICU stay, days	2.06 ± 1.32	2.36 ± 2.00	2.19 ± 1.81	2.21 ± 1.76	0.127
First episode ACS	215 (84.6%)	255 (85.0%)	320 (86.5%)	790 (85.5%)	0.779
Complications, % of patients	18 (7.1%)	38 (12.7%)	41 (11.1%)	97 (10.5%)	0.088
NYHA scale					
n	190	130	179	499	0.417
0	179 (94.2%)	125 (96.2%)	164 (91.6%)	468 (93.8%)	
1	9 (4.7%)	4 (3.1%)	12 (6.7%)	25 (5.0%)	
2	2 (1.1%)	1 (0.8%)	1 (0.6%)	4 (0.8%)	
3	0 (0.0%)	0 (0.0%)	2 (1.1%)	2 (0.4%)	
Killip					
n	242	287	354	883	0.095
I	226 (93.4%)	262 (91.3%)	312 (88.1%)	800 (90.6%)	
II	15 (6.2%)	24 (8.4%)	34 (9.6%)	73 (8.3%)	
III	0 (0.0%)	0 (0.0%)	5 (1.4%)	5 (0.6%)	
IV	1 (0.4%)	1 (0.3%)	3 (0.8%)	5 (0.6%)	

Data are n (%) or mean ± standard deviation. Comparisons between groups by ANOVA or chi-squared tests referring to the global distribution of each variable in the three study groups. * Significant *p*-values (*p* < 0.05). OSA: Obstructive sleep apnea; ACS: acute coronary syndrome; LVEF: left ventricular ejection fraction; ICU: intensive care unit; NYHA: New York Heart Association.

## Data Availability

All data generated or analyzed during this study are included in this published article.

## Abbreviations

ABSU	absorbance units
ACS	acute coronary syndrome
AHI	apnea hypopnea index
ALT	alanine aminotransferase
APTT	activated partial thromboplastin clotting time
ARAII	angiotensin II receptor antagonists
AST	aspartate aminotransferase
ACEI	angiotensin convertase enzyme inhibitors
BMI	body mass index
BP	blood pressure
CK	creatine kinase
CK-MB	creatine kinase isoform 2
CPAP	continuous positive airway pressure
CRP	C-reactive protein
CV	cardiovascular
EDS	excessive daytime sleepiness
EDTA	ethylene diamine tetra-acetic acid
ELISA	enzyme-linked immunosorbent assay
ESS	Epworth sleepiness scale
FDA	Food and Drug Administration
HBP	high blood pressure
HDL	high density lipoprotein
Hs-cTnT	high sensitive cardiac troponin T
ICU	intensive care unit
IL-6	interleukin 6
IL-8	interleukin 8
IQR	interquartile range
IMA	ischemia-modified albumin
LDL	low density lipoprotein
LVEF	left ventricular ejection fraction
Min SaO_2_	minimum nocturnal oxygen saturation
mDBP	median diastolic blood pressure
mSBP	median systolic blood pressure
N-terminal	amino terminal region
NYHA	New York Heart Association
OSA	obstructive sleep apnea
ODI	oxygen desaturation index
SaO_2_	oxygen saturation
SD	standard deviation
SEM	standard error of the mean
ROS	reactive oxygen species

## Appendix A

**Table A1:** Blood pressure and blood sample parameters in different groups.

Parameters	No/Mild OSA	Moderate OSA	Severe OSA	*p*-Value
mSBP, mm Hg	121.93 ± 17.26	122.85 ± 16.41	124.73 ± 18.36	0.122
mDBP, mm Hg	72.01 ± 11.31	71.76 ± 10.55	72.72 ± 10.60	0.491
Heart rate, bpm	72.25 ± 12.08	72.27 ± 12.53	72.64 ± 13.34	0.907
Hematocrit, %	42.40 ± 5.60	41.91 ± 4.43	42.27 ± 4.27	0.431
Hemoglobin, g/L	144.92 ± 16.34	142.20 ± 15.83	143.22 ± 15.72	0.133
Erythrocytes, ×10^12^/L	4.72 ± 0.55	4.67 ± 0.53	4.70 ± 0.52	0.568
Leukocytes, ×10^3^/µL	10.11 ± 3.17	10.12 ± 2.91	9.97 ± 2.78	0.774
Platelets, ×10^3^/µL	221.72 ± 61.15	215.69 ± 55.21	217.52 ± 63.12	0.489
Glucose, mg/dL	120.80 ± 50.32	125.05 ± 51.69	131.06 ± 54.39	0.052
Creatinine, mg/dL	1.13 ± 3.95	1.84 ± 8.49	2.96 ± 26.66	0.426
Uric acid, mg/dL	5.59 ± 2.54	5.96 ± 1.56	6.46 ± 5.16	0.047
Cholesterol, mg/dL	175.62 ± 41.75	178.57 ± 42.20	179.65 ± 43.87	0.523
HDL, mg/dL	41.66 ± 11.74	41.07 ± 11.22	40.79 ± 18.13	0.780
LDL, mg/dL	106.94 ± 37.60	107.74 ± 34.90	110.36 ± 35.79	0.487
Triglycerides, mg/dL	138.47 ± 79.56	149.04 ± 81.31	164.74 ± 117.05	0.005 *
AST, U/L	60.83 ± 64.05	78.17 ± 93.07	68.89 ± 86.43	0.082
ALT, U/L	35.73 ± 29.25	43.65 ± 130.86	38.74 ± 30.95	0.502

Data are mean ± standard deviation according to their distribution. * Significant *p*-values (*p* < 0.05). OSA: Obstructive sleep apnea; mSBP: mean systolic blood pressure; mDBP: mean diastolic blood pressure; bpm: beats per minute; HDL: high density lipoprotein; LDL: low density lipoprotein; AST: aspartate aminotransferase; ALT: alanine aminotransferase; CK: creatine kinase.
